# Repeat-containing protein effectors of plant-associated organisms

**DOI:** 10.3389/fpls.2015.00872

**Published:** 2015-10-21

**Authors:** Carl H. Mesarich, Joanna K. Bowen, Cyril Hamiaux, Matthew D. Templeton

**Affiliations:** ^1^School of Biological Sciences, The University of AucklandAuckland, New Zealand; ^2^Host–Microbe Interactions, Bioprotection, The New Zealand Institute for Plant & Food Research LtdAuckland, New Zealand; ^3^Human Responses, The New Zealand Institute for Plant & Food Research LimitedAuckland, New Zealand

**Keywords:** repeat-containing protein effectors, plant-associated organisms, microbes, nematodes, insects

## Abstract

Many plant-associated organisms, including microbes, nematodes, and insects, deliver effector proteins into the apoplast, vascular tissue, or cell cytoplasm of their prospective hosts. These effectors function to promote colonization, typically by altering host physiology or by modulating host immune responses. The same effectors however, can also trigger host immunity in the presence of cognate host immune receptor proteins, and thus prevent colonization. To circumvent effector-triggered immunity, or to further enhance host colonization, plant-associated organisms often rely on adaptive effector evolution. In recent years, it has become increasingly apparent that several effectors of plant-associated organisms are repeat-containing proteins (RCPs) that carry tandem or non-tandem arrays of an amino acid sequence or structural motif. In this review, we highlight the diverse roles that these repeat domains play in RCP effector function. We also draw attention to the potential role of these repeat domains in adaptive evolution with regards to RCP effector function and the evasion of effector-triggered immunity. The aim of this review is to increase the profile of RCP effectors from plant-associated organisms.

## Effectors of plant-associated organisms

Diverse plant-associated organisms, including bacteria, fungi, oomycetes, nematodes, and insects, secrete or inject a suite of proteins, termed effectors, into the tissues of their prospective hosts (Bozkurt et al., 2012; Deslandes and Rivas, 2012; Mitchum et al., 2013; Jaouannet et al., 2014; Lo Presti et al., 2015). These effectors, which localize to the host apoplast, or are targeted to various plant cell compartments, function to promote colonization, typically by altering host physiology or by modulating host immune responses (Hogenhout et al., 2009; Win et al., 2012a). Certain host plants however, have evolved immune receptor proteins that are capable of directly or indirectly recognizing one or more of these effectors or their modulated host targets respectively, to trigger immune responses that prevent colonization (Böhm et al., 2014; Cui et al., 2015). To circumvent these recognition events, or to provide novel, altered, or extended effector functionalities that further enhance the colonization of susceptible hosts, plant-associated organisms often rely on effector modification through adaptive evolution, as driven by host-imposed selection pressure (e.g., Stergiopoulos et al., 2007; Win et al., 2007; Dong et al., 2014).

## Several effectors of plant-associated organisms are repeat-containing proteins

Proteins that make up the effector repertoires of plant-associated organisms possess a range of different features. For example, most carry a signal peptide for targeted secretion or delivery to the host environment. In addition, many effectors, particularly those of fungi, are small and/or cysteine-rich, while others may possess a nuclear localization signal (NLS) or, as shown for several effectors of filamentous plant-associated organisms, a conserved effector motif (Dou and Zhou, 2012). The secretomes, and thus effector repertoires, of plant-associated organisms also differ in their proportion of repeat-containing proteins (RCPs). This is best illustrated by the predicted secretomes of *Melampsora larici-populina* and *Puccinia graminis* f. sp. *tritici*, the fungal pathogens responsible for poplar leaf rust and wheat stem rust, respectively. In a study by Saunders et al. (2012), it was revealed that of the 1549 secreted proteins predicted from the proteome of *M. larici-populina*, 493 (~32%) were RCPs. In contrast, no RCPs could be identified among the 1852 secreted proteins predicted from the proteome of *P. graminis* f. sp. *tritici* (Saunders et al., 2012). As such, RCP effectors are expected to play an important role in promoting the colonization of some, but not all, plant-associated organisms. This is supported by the fact that several known effectors of plant-associated organisms are RCPs (Tables 1–3). For the purpose of this review, we define RCPs as those proteins that carry two or more copies of a tandemly or non-tandemly duplicated sequence or structural motif that is at least five amino acid residues in length.

**Table 1:** **Examples of repeat-containing protein (RCP) effectors from plant-associated bacteria**.

**RCP (aa^a^)**	**Plant-associated organism**	**Host plant (relationship with host)**	**Repeat features^b^**	**Localization *in planta***	**Part of an RCP effector family?**	**References**
AvrPtoB/HopAB2 (553)	*Pseudomonas syringae* pv. *tomato* (plant-pathogenic bacterium)	Tomato (causes speck disease)	Two amphipathic degenerate non-tandem repeats of 85 and 110 aa identified by structural analyses that each adopt a four-helix bundle fold	Host cell cytoplasm	No	Kim et al., 2002; Abramovitch et al., 2003, 2006; de Torres et al., 2006; He et al., 2006; Janjusevic et al., 2006; Mucyn et al., 2006; Rosebrock et al., 2007; Xiao et al., 2007; Göhre et al., 2008; Shan et al., 2008; Dong et al., 2009; Gimenez-Ibanez et al., 2009; Cheng et al., 2011; Zeng et al., 2012; Mathieu et al., 2014
Biological function: AvrPtoB, a type III effector that suppresses host immunity, carries an amino (N)-terminal and central repeat unit (repeat units one and two, respectively), as well as a carboxyl (C)-terminal U-box-type E3 ubiquitin ligase domain. Repeat units one and two bind and inhibit the kinase domain of the plasma membrane (PM)-localized host lysin motif (LysM)-receptor-like kinase (RLK) and leucine-rich repeat (LRR)-RLK immune receptors, Bti9 and BAK1, respectively, to suppress immunity related signaling. Repeat units one and two also bind the kinase domain of the LysM-RLK CERK1 and LRR-RLK FLS2 immune receptors, respectively, which may promote their ubiquitination and subsequent proteasome-dependent degradation via the E3 ligase domain. In addition, repeat unit one interacts with the host receptor-like cytoplasmic kinase (RLCK), Pto, while repeat unit two interacts with Pto and a related host RLCK, Fen. Following interaction with AvrPtoB, Pto activates host immunity in conjunction with Prf, an immune receptor of tomato. Fen however, can only activate host immunity in the absence of the E3 ubiquitin ligase domain. Interaction of Pto or Fen with repeat unit two results in the proteasome-dependent degradation of these proteins as above. Pto however, is able to resist degradation to activate Prf-dependent immunity upon interaction with repeat unit one, as this repeat unit is further away from the E3 ubiquitin ligase domain						
HopI1 (432)	*Pseudomonas syringae* pv. *maculicola* (plant-pathogenic bacterium)	Brassicaceae (causes leaf spot disease)	Four hydrophilic imperfect intrinsically disordered tandem proline and glutamine (P/Q)-rich repeats of 27, 37, or 38 aa	Host cell chloroplast	No	Guttman et al., 2002; Jelenska et al., 2007, 2010
Biological function: HopI1 is a type III effector that carries an N-terminal region of unknown function, a central repeat domain, and a C-terminal J-domain. HopI1 suppresses salicylic acid (SA) accumulation and related plant defenses. HopI1 also induces the remodeling of thylakoid stacks within chloroplasts. The J-domain of HopI1 directly binds to different plant Hsp70 isoforms and stimulates Hsp70 ATP hydrolysis activity *in vitro*. In association with Hsp70, HopI1 forms large complexes *in planta*, and recruits cytosolic Hsp70 to chloroplasts, a requirement for its virulence function. It has been suggested that Hsp70 may affect the folding/complex assembly of chloroplast factors related to plant immunity, including those required for SA biosynthesis and transport. The HopI1 repeat domain is not required for the interaction with Hsp70 or the association of this effector with chloroplasts. However, it is required for HopI1 virulence function. Thus, the HopI1 repeat domain may for example, interfere with these processes by actively affecting Hsp70 activity and/or substrate specificity						
HsvG (671)	*Pantoea agglomerans* pv. *gypsophilae* (plant-pathogenic bacterium)	Gypsophila (root and crown gall disease)	Two amphipathic imperfect tandem repeats of 75 and 71 aa	Host cell nucleus	Yes	Valinsky et al., 1998; Nissan et al., 2006, 2012
Biological function: HsvG is a type III effector that carries a central DNA-binding region and repeat domain (transcription activation domain; TAD). HsvG functions as a transcription factor that binds and activates the *HSVGT* gene promoter from *Gypsophila paniculata*. *HSVGT* encodes a predicted protein of the DnaJ family that has features typical of eukaryotic transcription factors, but lacks a J-domain. In addition to transcriptional activation, the HsvG repeat domain is required for host specificity (*P. agglomerans* pv. *gypsophilae* pathogenicity on gypsophila). HsvG requires two repeat units for pathogenicity on gypsophila (one is not sufficient)						
PthXo1 (1373)	*Xanthomonas oryzae* pv. *oryzae* (plant-pathogenic bacterium)	Rice (causes blight disease)	Four amphipathic degenerate tandem repeats of 25 or 34 aa, followed by 23.5 amphipathic imperfect tandem repeats of 33 or 34 aa. All repeat units adopt a two-helix bundle fold	Host cell nucleus	Yes	Yang and White, 2004; Chu et al., 2006; Yang et al., 2006; Yuan et al., 2009; Chen et al., 2010; Gao et al., 2012; Mak et al., 2012
Biological function: PthXo1 is a type III transcription activator-like (TAL) effector that binds and transcriptionally activates the promoter of *OsSWEET11*, a susceptibility gene from rice that encodes a SWEET sugar transporter, to promote colonization. It is thought that OsSWEET11 expression results in an excess of sucrose transport to the site of infection by *X. oryzae* pv. *oryzae*, which in turn provides the pathogen with a source of carbon. PthXo1 carries a central repeat domain, which forms a left-handed superhelix (α-solenoid) that physically wraps around the effector-binding element (EBE) of *OsSWEET11*, as well as a C-terminal eukaryotic activation domain (AD), which induces *OsSWEET11* transcription. The first two degenerate repeat units of PthXo1 mediate non-base-specific interactions with the EBE, while the two subsequent degenerate repeat units mediate pairing with the EBE's initial 5′ thymine base. The remaining imperfect repeat units mediate base-specific interactions with the EBE, with specificity determined by the repeat-variable di-residues (RVDs) at positions 12 and 13 of each repeat unit. The recessive *OsSWEET11* allele, *xa13*, confers resistance to *X. oryzae* pv. *oryzae*, and is based on a naturally mutated EBE that can neither be bound nor transcriptionally activated by PthXo1						
RipG7/GALA7 (647)	*Ralstonia solanacearum* (plant-pathogenic bacterium)	Broad host range (causes wilt disease)	Fifteen amphipathic degenerate mostly tandem LRRs of ~21–25 aa	Intracellular (host)	Yes	Cunnac et al., 2004; Angot et al., 2006; Remigi et al., 2011; Wang et al., 2015a
Biological function: RipG7 is a type III effector that carries an N-terminal F-box domain followed by a LRR domain. RipG7 interacts with several *Arabidopsis thaliana* SKP1-like (ASK) proteins. Together with six of its paralogs (RipG1–RipG6), RipG7 is essential for pathogenicity on *A. thaliana*, although functionally redundant with RipG2, 3 and 6, and required for full virulence on tomato. RipG7 is a virulence factor required for host-specific colonization of *Medicago truncatula*, with the F-box being essential for virulence, suggesting that RipG7 may mimic host F-box proteins and be recruited to SCF-type E3 ubiquitin ligase complexes to interfere with host ubiquitination and proteasome processing. The LRR domain is expected to recruit specific plant proteins to a SCF^RipG7^ E3 ubiquitin ligase for subsequent ubiquitination and possible degradation. Ten of 11 amino acid residue sites identified as being under strong positive selection across RipG7 from phylogenetically diverse strains of *R. solanacearum* are located within, or in loops between, predicted LRRs. This suggests an evolutionary arms race between *R. solanacearum* and its hosts that occurs at the interaction interface between RipG7 and its putative host targets						
RipL (1390)	*R. solanacearum*		Five amphipathic degenerate tandem pentatricopeptide repeats (PPRs) of 35 aa^c^	Intracellular (host)	No	Cunnac et al., 2004
Biological function: RipL is a type III effector with unknown function. PRRs possibly mediate the binding of RNA						
RipS4/SKWP4 (2574)	*R. solanacearum*		At least 18 amphipathic imperfect/degenerate tandem HEAT/armadillo repeats of 40–42 aa	Intracellular (host)	Yes	Mukaihara and Tamura, 2009; Macho et al., 2010
Biological function: RipS4 is a type III effector required for full virulence on eggplant, although its specific function is unknown						
RipTAL1 (1245)	*R. solanacearum*		Two amphipathic degenerate tandem repeats of 34 or 35 aa, followed by 16 amphipathic imperfect tandem repeats of 35 aa, and two amphipathic degenerate tandem repeats of 34 aa	Host cell nucleus	No	Macho et al., 2010; de Lange et al., 2013; Li et al., 2013
Biological function: RipTAL1 is a type III TAL effector required for full virulence of *R. solanacearum* on eggplant, and probably promotes virulence through the transcriptional activation of a host susceptibility gene. RipTAL1 carries a central repeat domain, which mediates interaction with the EBE of a target host gene promoter, and a C-terminal eukaryotic acidic AD, which induces transcription of the target host gene. The N-terminal degenerate repeat units of RipTAL1 mediate pairing with EBEs containing an initial 5′ guanine base. The imperfect repeat units mediate base-specific interactions with the EBE, with specificity mainly determined by RVDs at positions 12 and 13 of each repeat unit, although certain non-RVD residues also have a significant impact on DNA recognition						
RipY (912)	*R. solanacearum*		At least six amphipathic degenerate mostly tandem ankyrin repeats of 31 aa^c^	Intracellular (host)	No	Cunnac et al., 2004; Macho et al., 2010
Biological function: RipY is a type III effector required for full virulence on eggplant, although its specific function is unknown						
XopAC/AvrAC (536)	*Xanthomonas campestris* pv. *campestris*	Brassicaceae (causes black rot disease)	Six amphipathic degenerate tandem LRRs of 23–24 aa	Intracellular (host PM)	No	Xu et al., 2008; Feng et al., 2012; Guy et al., 2013; Wang et al., 2015b
Biological function: XopAC is a type III effector that enhances the virulence of *X. campestris* pv. *campestris* and suppresses plant immunity. It has an N-terminal region, followed by a LRR domain, and a C-terminal FIC (filamentation induced by cyclic AMP) domain, with the latter possessing uridine 5′-monophosphate (UMP) transferase enzymatic activity. In susceptible *A. thaliana* plants, the FIC domain transfers UMP to phosphorylation sites in the activation loop of several immunity-related RLCKs, including BIK1, PBL1, and RIPK. This prevents their phosphorylation, thereby reducing their kinase activities and inhibiting their downstream immune signaling. In *A. thaliana* ecotype Col-0 vascular tissues, XopAC is recognized as an avirulence protein, with the LRRs, as well as the FIC domain and its associated uridylylation activity required to trigger the avirulent phenotype. Such immunity is dependent upon the RLCK, PBL2. Although PBL2 is a paralog of BIK1, it is solely required for immunity, indicating that PBS2 is a decoy of BIK1 that enables XopAC recognition by the host. Notably, the N-terminal region and LRR domain of XopAC are required for the interaction with RLCKs, with localization of XopAC to the host PM also dependent upon the LRRs						
XopD (760)	*Xanthomonas euvesicatoria* (plant-pathogenic bacterium)	Tomato/pepper (causes leaf spot disease)	Two amphipathic tandem ERF-associated repression (EAR) motifs of 6 aa	Host cell nucleus (subnuclear foci)	No	Hotson et al., 2003; Chosed et al., 2007; Kim et al., 2008, 2013
Biological function: XopD is a type III effector that promotes pathogen growth by suppressing activation of host immunity via plant SUMO protease mimicry. It has an N-terminal DNA-binding domain (DBD), two EAR motifs (typically found in plant repressors that regulate stress-induced transcription) in the central domain and a C-terminal SUMO peptidase domain. XopD possesses both plant-specific peptidase activity, resulting in cleavage of SUMO isoforms, and isopeptidase activity, resulting in cleavage of SUMO from SUMO conjugates. All three domains are collectively required to desumoylate the transcription factor SIERF4 to suppress ethylene production and signaling. The mechanism by which the DBD and EAR motifs modulate the protease activity is not known, however they may mediate critical interactions with DNA or proteins within plant transcription factor complexes to influence effector specificity						
XopL (660)	*X. euvesicatoria*		Nine amphipathic degenerate tandem LRRs of 23–33 aa	Intracellular (host)	No	Singer et al., 2013
Biological function: XopL is a type III effector that has E3 ubiquitin ligase activity responsible for initiating cell death in the non-host *Nicotiana benthamiana*, but not in the hosts tomato and pepper. The N-terminal LRR domain is solely required for host immunity-related gene expression when assayed in *A. thaliana*. XopL recruits plant E2 enzymes, mimicking components of the host ubiquitination machinery, with the LRRs possibly acting as protein–protein interaction modules for ubiquitination target recognition						
XopN (733)	*X. euvesicatoria*		Seven amphipathic degenerate tandem HEAT/armadillo-like repeats	Host cytoplasm and PM	No	Roden et al., 2004; Kim et al., 2009; Taylor et al., 2012
Biological function: XopN is a type III effector that suppresses host immune responses. It interacts with the atypical LRR-RLK, TARK1 (via the non-repetitive N-terminal region), and the tomato 14-3-3 isoform TFT1 (via the C-terminal HEAT/armadillo-like repeats), both of which are positive regulators of host immunity in tomato. XopN is expected to promote and/or stabilize TARK1/TFT1 complex formation by functioning as a protein bridge or molecular scaffold, since these proteins only interact in the presence of XopN. It remains unclear how these interactions repress the host immune response, although XopN may interfere with TARK1 protein–protein interactions, stability and/or signal transduction, and TFT1 client interactions. Another possibility is that the action of XopN leads to the sequestration of inactive immune complexes, preventing downstream immune signaling						

a *Protein length in amino acids (aa)*.

b *Repeat hydropathy profiles were determined using the Expasy ProtScale server (http://web.expasy.org/protscale/), with default server settings*.

c *PPR and ankyrin repeats were predicted using TPRpred (http://toolkit.tuebingen.mpg.de/tprpred) and InterProScan 5 (http://www.ebi.ac.uk/Tools/pfa/iprscan5/), respectively*.

**Table 2:** **Examples of repeat-containing protein (RCP) effectors and surface-associated RCPs from plant-associated fungi and oomycetes**.

**RCP (aa^a^)**	**Plant-associated organism**	**Host plant (relationship with host)**	**Repeat features^b^**	**Localization *in planta***	**Part of an RCP effector family?**	**References**
ATR1^Emoy2^ (311)	*Hyaloperonospora arabidopsidis* (plant-pathogenic oomycete)	*Arabidopsis thaliana* (causes downy mildew disease)	Two amphipathic degenerate tandem WY domain repeats of 83 and 100 aa identified by structural analysis that adopt a five-helix bundle fold	Host cell cytoplasm	Yes	Rehmany et al., 2005; Sohn et al., 2007; Krasileva et al., 2010; Chou et al., 2011; Steinbrenner et al., 2015
Biological function: ATR1 from isolate Emoy2 (ATR1^Emoy2^) contributes to pathogen virulence, although its specific function is unknown. ATR1^Emoy2^ is directly recognized by the RPP1^NdA^ and RPP1^WsB^ immune receptors of *A. thaliana*. One and two aa residues in ATR1^Emoy2^ associated with the recognition of this effector by RPP1^NdA^ and RPP1^WsB^, respectively, are located on the surface of repeat unit one. Other aa residues, as identified by gain-of-(RPP1^NdA^)-recognition mutagenesis screens using traditionally non-recognized ATR1 alleles are also located on the surface of repeat unit one						
ATR13 (187)	*H. arabidopsidis*		Six hydrophilic degenerate tandem leucine/isoleucine repeats of 7 aa, followed by 4 hydrophilic imperfect tandem repeats of 11 aa. Repeats are located in a disordered region of the protein	Host cell nucleolus	No	Allen et al., 2004, 2008; Sohn et al., 2007; Leonelli et al., 2011
Biological function: ATR13 contributes to pathogen virulence, possibly by suppressing host immune responses, although its specific function is unknown. ATR13 is recognized by the RPP13^Nd^ immune receptor of *A. thaliana*. Mutations cannot be made to particular leucine or isoleucine residues within the 7-aa repeats of ATR13 without altering recognition by RPP13^Nd^. The 11-aa repeats of ATR13 are required for nucleolar localization. Alleles of ATR13 carrying only one of the four 11-aa repeats do not localize to the nucleolus. However, when the three missing repeats are added to these alleles, nucleolar localization is observed						
AvrM-A (343)	*Melampsora lini* (plant-pathogenic fungus)	Flax (causes leaf rust disease)	Two hydrophilic degenerate tandem repeats of 68 aa identified by structural analysis that adopt a four-helix bundle fold. Repeats share some similarity in the overall architecture to the WY domain of several oomycete effectors	Host cell cytoplasm	Yes	Catanzariti et al., 2006, 2010; Rafiqi et al., 2010; Ve et al., 2013
Biological function: AvrM-A function unknown. AvrM is directly recognized by the M immune receptor of flax. Several residues of AvrM required for recognition by M are located on the surface of repeat unit two						
CBEL (268)	*Phytophthora parasitica* var. *nicotianae* (plant-pathogenic oomycete)	Tobacco (causes black shank root and crown rot disease)	Two amphipathic imperfect near-tandem repeats of 113 and 114 aa comprising a carbohydrate-binding module family 1 (CBM1)/fungal-type cellulose-binding domain (CBD) linked to a PAN/APPLE domain	Cyst surface and hyphal cell wall	No	Séjalon-Delmas et al., 1997; Villalba Mateos et al., 1997; Gaulin et al., 2002, 2006; Khatib et al., 2004
Biological function: the CBDs of CBEL play a role in the adhesion of mycelia to cellulosic substrates, including plant cell walls, and in the organized deposition of the *P. parasitica* var. *nicotianae* cell wall polysaccharide, β-glucan. However, knockdown transformants do not display significantly reduced virulence. CBEL also elicits strong host immune responses when infiltrated into tobacco, as well as various non-host plants, including *A. thaliana*. These immune responses require the binding of CBEL to the plant cell wall, as mediated through the CBDs						
Cin1^c^ (523)	*Venturia inaequalis* (plant-pathogenic fungus)	Apple (causes scab disease)	Eight hydrophilic imperfect tandem repeats of 52–64 aa that adopt a core helix-loop-helix motif as part of a three-helix bundle fold	Unknown	Yes	Kucheryava et al., 2008; Mesarich et al., 2012
Biological function: Cin1 function unknown. *Cin1* gene expression is induced *in planta*						
CTP1 (171)	*Melampsora larici-populina* (plant-pathogenic fungus)	Poplar (causes leaf rust disease)	Two amphipathic imperfect near-tandem repeats of 64 aa	Host cell chloroplast (stroma) and mitochondria	Yes	Petre et al., 2015a,b
Biological function: CTP1 function unknown. Repeat unit one overlaps with a predicted chloroplast transit peptide						
Ecp6 (222)	*Cladosporium fulvum* (plant-pathogenic fungus)	Tomato (causes leaf mold disease)	Three amphipathic degenerate near-tandem lysin motif (LysM) domain repeats of 44 or 45 aa that adopt a β*ααβ*-fold	Host apoplast	No	Bolton et al., 2008; de Jonge et al., 2010; Thomma et al., 2011; Sánchez-Vallet et al., 2013
Biological function: Ecp6 perturbs chitin-triggered immunity in tomato by sequestering chitin oligosaccharides released from the fungal cell wall. More specifically, LysM1 and LysM3 domain repeats out-compete host chitin receptors for the binding of chitin oligosaccharides. The LysM2 domain repeat may perturb chitin-triggered immunity through a yet unknown mechanism. Ecp6 is recognized by the Cf-Ecp6 immune receptor of tomato						
Hum3 (828)	*Ustilago maydis* (plant-pathogenic fungus)	Maize (causes smut disease)	Seventeen amphipathic imperfect tandem repeats of 31–36 aa. Fourteen repeats are separated by putative Kex2 processing motifs	Unknown	No	(Teertstra et al., 2006; Müller et al., 2008)
Biological function: Hum3 function unknown. Deletion of *Hum3* alone does not affect virulence of *U. maydis* on maize. However, the pathogenic development of a *Hum3*/*Rsp1* double knock-out mutant is halted *in planta* shortly after penetration. The Hum3 repeat domain is followed by a hydrophobin domain						
Rep1 (652)	*U. maydis*		Twelve amphipathic imperfect mostly tandem repeats of 34–55 aa. Repeats are separated by Kex2 sites, and are proteolytically processed into 10 small amphipathic peptides (Rep1-1–Rep1-10) of 35–53 aa, and one of 228 aa (Rep1-11)	Hyphal cell wall	No	Wösten et al., 1996; Teertstra et al., 2006, 2009; Müller et al., 2008
Biological function: Rep1 is a repellent protein. Following the proteolytic processing of Rep1 by Kex2, processed repellent peptides form surface-active amyloid-like fibrils at the hyphal surface that play a role in cellular attachment to hydrophobic surfaces (e.g., the host surface) and in the formation of aerial hyphae. Rep1 does not appear to be required for the virulence of *U. maydis* on maize						
Rsp1 (260)	*U. maydis*		Eleven hydrophilic imperfect tandem repeats of 18 or 21 aa. Repeats are separated by putative Kex2 processing motifs	Unknown	No	Müller et al., 2008
Biological function: Rsp1 function unknown. Deletion of *Rsp1* alone does not affect virulence of *U. maydis* on maize. However, the pathogenic development of a *Hum3*/*Rsp1* double knock-out mutant is halted *in planta* shortly after penetration						
SP7 (499)^d^	*Rhizophagus irregularis* (arbuscular mycorrhizal fungus)	Broad host range (mutualistic symbiont of plant roots)	Up to 10 hydrophilic imperfect repeats of 6–16 aa, separated by four hydrophilic imperfect repeats of 7 or 8 aa	Host cell nucleus	Yes	Kloppholz et al., 2011
Biological function: SP7 interacts with the pathogenesis-related ethylene-responsive host transcription factor ERF19 to promote symbiotic biotrophy. Possibly counteracts ERF19-regulated host defense responses						

a *Protein length in amino acids (aa)*.

b *Repeat hydropathy profiles were determined using the Expasy ProtScale server (http://web.expasy.org/protscale/), with default server settings*.

c *Cin1 is a candidate effector of V. inaequalis (Kucheryava et al., 2008)*.

d *The length of SP7 remains unclear due to differential transcript splicing, with five versions of the mRNA transcript found at different developmental stages (Kloppholz et al., 2011)*.

**Table 3:** **Examples of repeat-containing protein (RCP) effectors from plant-associated nematodes and insects**.

**RCP (aa^a^)**	**Plant-associated organism**	**Host plant (relationship with host)**	**Repeat features^b^**	**Localization *in planta***	**Part of an RCP effector family?**	**References**
Gp-HYP (varies^c^)	*Globodera pallida* (plant-parasitic nematode)	Solanaceae (sedentary endoparasite of plant roots)	Three Gp-HYP effector subfamilies (Gp-HYP-1, Gp-HYP-2, and Gp-HYP-3) containing a various number of hydrophilic perfect and imperfect tandem repeats of 5–17 aa	Host apoplast	Yes	Eves-van den Akker et al., 2014
Biological function: Gp-HYP effectors contribute to *G. pallida* parasitism, although their specific functions are unknown						
GrCLE1 (204)	*Globodera rostochiensis* (plant-parasitic nematode)	Solanaceae (sedentary endoparasite of plant roots)	Four hydrophilic imperfect CLAVATA3 (CLV3)/endosperm surrounding region (ESR) (CLE)-like motif repeats of 12 aa (three identical), separated by a hydrophilic imperfect spacer repeat of 9 aa	Host apoplast	Yes	Lu et al., 2009; Guo et al., 2011; Chen et al., 2015
Biological function: GrCLE1 is processed into at least three arabinosylated CLE-like peptides by host proteases. These CLE-like peptides directly bind plant receptor-like kinases (RLKs), including CLV2, BAM1, and BAM2, where they function as endogenous plant CLE peptide mimics to incite changes in plant root growth and development that facilitate parasitism						
MAP (varies^c^)	*Meloidogyne incognita* (plant-parasitic nematode)	Broad host range (sedentary endoparasite of plant roots)	Up to nine hydrophilic imperfect CLE-like motif repeats of 14 aa. A hydrophilic imperfect *Heterodera* variable domain-like motif (HVLM) repeat of 15 aa is often interspersed between CLE-like motifs	Host apoplast	Yes	Semblat et al., 2001; Castagnone-Sereno et al., 2009; Vieira et al., 2011; Rutter et al., 2014
Biological function: MAP effectors are possibly processed into CLE-like peptides that function as mimics of endogenous plant CLE peptides. These peptides then possibly interact with cognate host RLKs to incite changes in plant root growth and development that facilitate parasitism. HVLM repeats may function in the trafficking of MAP effectors into the host apoplast, the processing of MAP effectors to mature CLE-like peptides, and/or host specificity. MAP-1 may be recognized by the Mi-1 immune receptor of tomato, with the number/arrangement of repeats in MAP-1 correlated with avirulence of *M. incognita*						
MpC002 (265)	*Myzus persicae* (aphid; plant pest)	Broad host range (phloem feeder)	Five hydrophilic perfect tandem repeats of 7 aa	Host vascular tissue (phloem)?	No	Bos et al., 2010; Pitino et al., 2011; Pitino and Hogenhout, 2013; Elzinga et al., 2014
Biological function: MpC002 increases *M. persicae* fecundity on *Arabidopsis thaliana, Nicotiana benthamiana*, and *Nicotiana tabacum* through an unknown mechanism. The repeat domain is required for this increased fecundity						
SSGP-71 (varies^c^)	*Mayetiola destructor* (Hessian fly; plant pest)	Cereals (gall-forming pest)	Typically 13 amphipathic degenerate tandem leucine-rich repeats (LRRs) of ~20–30 aa	Likely intracellular (host)	Yes	Zhao et al., 2015
Biological function: SSGP-71 effectors typically contain an amino (N)-terminal cyclin-like F-box, followed by carboxyl (C)-terminal LRRs. These effectors, which interact with host Skp proteins, are suspected to mimic host F-box-LRR proteins in order to hijack the plant proteasome for the purpose of directly producing nutritive tissue, defeating plant immunity, and/or stunting plant growth. The LRR domain of SSGP-71 effectors is expected to provide target (host protein) specificity. The SSGP-71 effectors Mdes009086-RA and Mdes015365-RA are recognized by the H6 and H9 immune receptors of wheat, respectively. Unlike Mdes009086-RA, Mdes015365-RA does not possess an F-box						
vH13 (116)	*M. destructor*		Three hydrophilic imperfect tandem repeats of 12 or 14 aa	Unknown	No	Aggarwal et al., 2014
Biological function: vH13 function unknown. vH13 is recognized by the H13 immune receptor of wheat						

a *Protein length in amino acids (aa)*.

b *Repeat hydropathy profiles were determined using the Expasy ProtScale server (http://web.expasy.org/protscale/), with default server settings*.

c *The protein length varies between members of the RCP effector family*.

**Figure 1:**
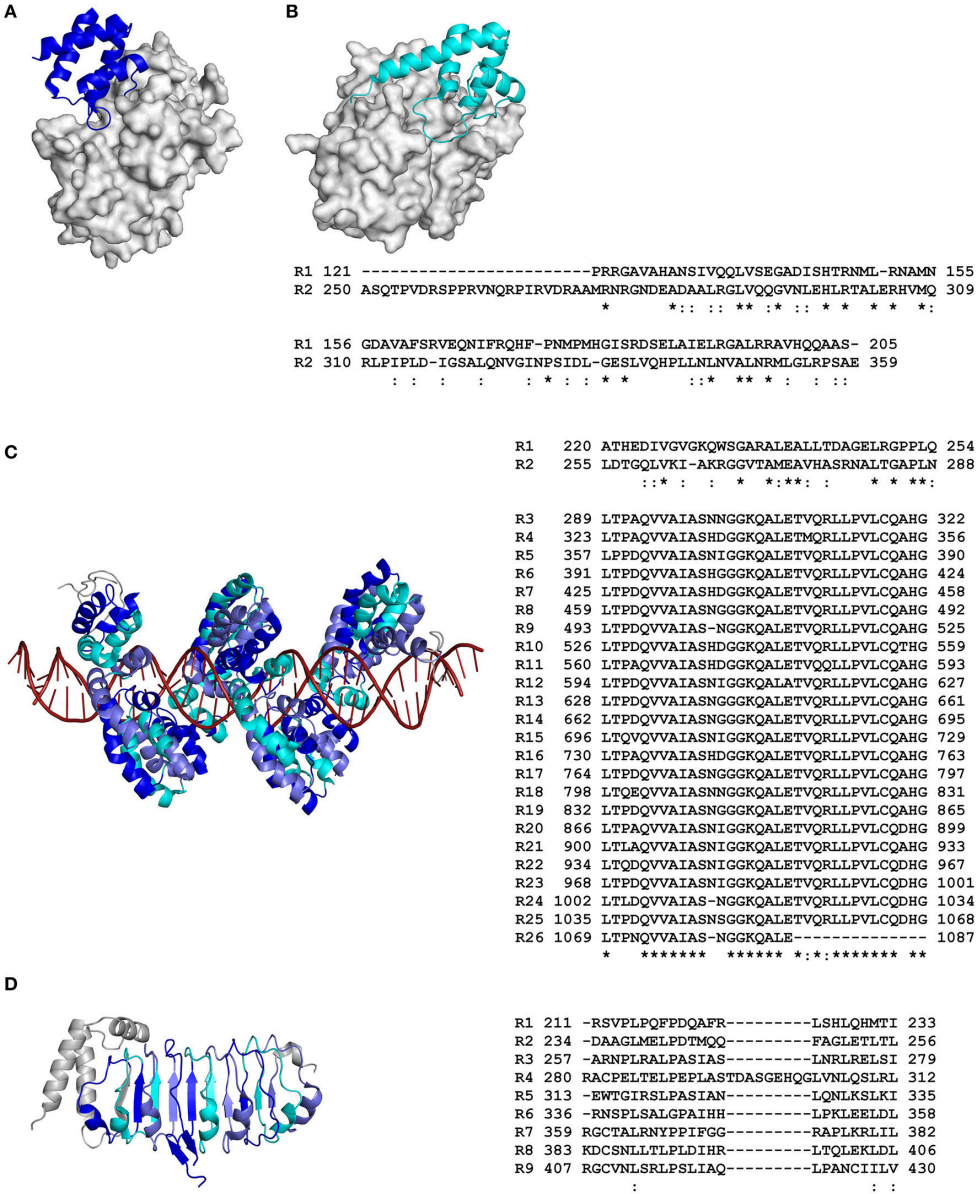
**Primary and tertiary structures of repeat domains from RCP effectors of plant-associated bacteria**. **(A)** Crystal structure of repeat unit one from the AvrPtoB effector of the tomato bacterial speck pathogen, *Pseudomonas syringae* pv. *tomato* (*Pst*), in complex with the tomato Pto kinase (Protein Data Bank [PDB] code 3HGK; Dong et al., 2009). **(B)** Nuclear magnetic resonance (NMR) structure of repeat unit two from AvrPtoB of *Pst* in complex with the BAK1 kinase domain from *Arabidopsis thaliana* (3TL8; Cheng et al., 2011). Note that in **(A)**, AvrPtoB repeat unit one interacts with the Pto kinase in a different orientation to that of AvrPtoB repeat unit two with the BAK1 kinase domain in **(B)**. **(C)** Crystal structure of the repeat domain from the PthXo1 transcription activator-like (TAL) effector of the bacterial rice pathogen, *Xanthomonas oryzae* pv. *oryzae*, bound to its natural DNA target (36 bp). The repeats pack together to form a left-handed superhelix (α-solenoid) that wraps around the DNA molecule (3UGM; Mak et al., 2012). **(D)** Crystal structure of the N-terminal leucine-rich repeat (LRR) domain from the XopL effector of the bacterial leaf spot pathogen of pepper and tomato, *Xanthomonas euvesicatoria* (4FCG; Singer et al., 2013). Structural coordinate files were downloaded from the Research Collaboratory for Structural Bioinformatics (RCSB) PDB (http://www.rcsb.org/pdb/home/home.do). Alternating repeat units are colored blue, slate, and cyan, respectively. Non-repetitive sequence is colored gray. The molecular surface of Pto kinase in **(A)** and BAK1 kinase domain in **(B)** are shown in gray, while the DNA molecule in **(C)** is colored red. An amino acid sequence alignment detailing the primary structure of each RCP effector repeat domain is shown to the right of each tertiary structure (as based on that presented in each tertiary structure). Repeat (R) units are numbered according to their position in the RCP effector. The start and end position of each repeat unit in the full-length RCP effector is shown. Conserved (^*^) and strongly similar (:) amino acid residues shared between repeat units are shown below the sequence alignment (based on full-length repeat units only). The figure was prepared using PyMol (https://www.pymol.org/) and Clustal Omega (http://www.ebi.ac.uk/Tools/msa/clustalo/).

**Figure 2:**
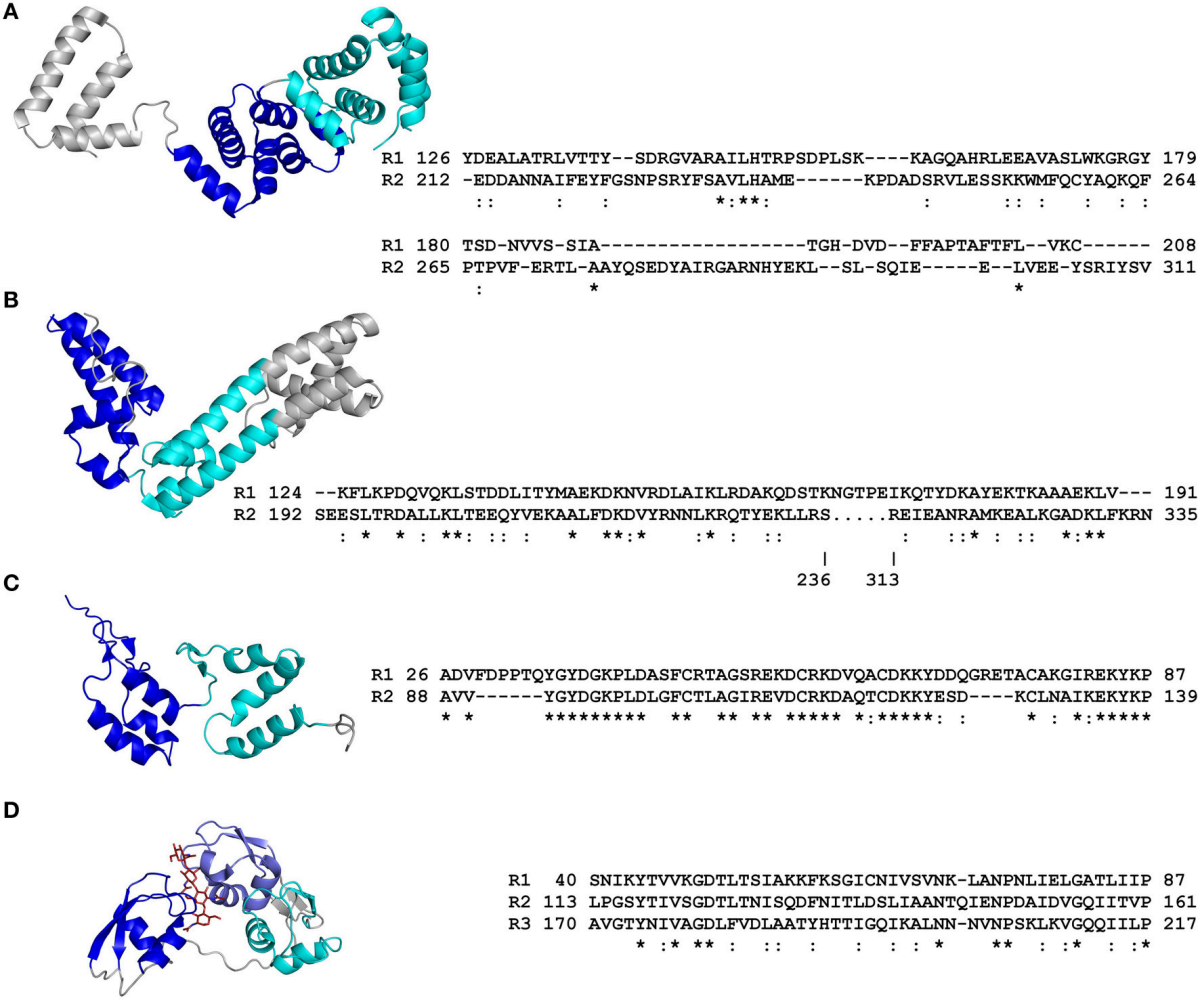
**Primary and tertiary structures of repeat domains from RCP effectors of plant-associated fungi and an oomycete**. **(A)** Crystal structure of the ATR1 effector from the *Arabidopsis thaliana* oomycete pathogen, *Hyaloperonospora arabidopsidis* (Protein Data Bank [PDB] code 3RMR; Chou et al., 2011). **(B)** Crystal structure of the AvrM-A effector from the flax rust fungus, *Melampsora lini* (4BJN; Ve et al., 2013). **(C)** Nuclear magnetic resonance (NMR) structure of repeat units 1 and 2 from the candidate effector Cin1 of the apple scab fungus, *Venturia inaequalis* (2LHT; Mesarich et al., 2012). **(D)** Crystal structure of the Ecp6 effector from the tomato leaf mold fungus, *Cladosporium fulvum*. The lysin motif (LysM) repeat units 1 and 3 coordinate the binding of a single chitin tetramer by means of an inter-repeat domain groove (4B8V; Sánchez-Vallet et al., 2013). Structural coordinate files were downloaded from the Research Collaboratory for Structural Bioinformatics (RCSB) PDB (http://www.rcsb.org/pdb/home/home.do). Alternating repeat units are colored blue, slate, and cyan, respectively. Non-repetitive sequence is colored gray. The chitin tetramer in **(D)** is colored red. An amino acid sequence alignment detailing the primary structure of each RCP effector repeat domain is shown to the right of each tertiary structure (as based on that presented in each tertiary structure). Repeat (R) units are numbered according to their position in the RCP effector. The start and end position of each repeat unit in the full-length RCP effector is shown. Conserved (^*^) and strongly similar (:) amino acid residues shared between repeat units are shown below the sequence alignment. The figure was prepared using PyMol (https://www.pymol.org/) and Clustal Omega (http://www.ebi.ac.uk/Tools/msa/clustalo/). Structure-based sequence alignments of repeat units from ATR1 and AvrM-A are adapted from Chou et al. (2011) and Ve et al. (2013), respectively.

Various bioinformatic tools, databases, and servers are available for the detection of repeat domains in protein sequences (reviewed in Kajava, 2012; Luo and Nijveen, 2014). Typically, perfect (identical) or imperfect (near-identical) sequence repeats are easily detected, as are those repeats with homology to known functional domains. However, the detection of highly degenerate (divergent) sequence repeats, which carry amino acid substitutions, insertions, or deletions that have accumulated during evolution, is often more difficult. In some instances, degenerate sequence repeats may only be identified following an analysis of protein tertiary structure, for which servers are again available (see Kajava, 2012). Indeed, this has been the case for several effectors of plant-associated organisms. As an example of this, structural characterization of both the AvrM-A effector from *Melampsora lini*, a fungal rust pathogen of flax, as well as AvrPtoB, a type III effector from *Pseudomonas syringae* pv. *tomato* (*Pst*), the bacterial speck pathogen of tomato, revealed the presence of two four-helix bundle repeats (Figures 1A,B, 2B) (Dong et al., 2009; Cheng et al., 2011; Ve et al., 2013). Bioinformatic tools though, have been shown to play a key role in the identification of certain highly degenerate repeat domains. For example, Jiang et al. (2008) used the MEME algorithm (Bailey et al., 2015), together with hidden Markov model (HMM) searches, to identify RXLR effectors from two plant-associated oomycete species (*Phytophthora sojae* and *Phytophthora ramorum*) that carry conserved, but highly degenerate, C-terminal WYL motifs, or WY motifs, which often form tandem repeats. In oomycete plant pathogens, RXLR effectors represent one of the largest and most diverse effector families (Jiang et al., 2008). Jiang et al. (2008) demonstrated that approximately half of the abovementioned RXLR effectors possess WYL motifs, with 30% possessing between two and eight repeated WYL modules. A comparison of RXLR effector tertiary structures has since revealed that a three-helix bundle fold, termed the WY domain, is the basic structural unit adopted by the WY motifs (Boutemy et al., 2011; Win et al., 2012b). One of these structurally characterized RXLR effectors, ATR1, which is produced by *Hyaloperonospora arabidopsidis*, the oomycete downy mildew pathogen of *Arabidopsis thaliana*, carries two five-helix bundle WY domain repeats (Figure 2A) (Chou et al., 2011). Notably though, this tandem repeat was only identified upon structural characterization of ATR1, with a prior HMM-based bioinformatic screen identifying only one of the two WY domains present in this effector (Boutemy et al., 2011). This example therefore highlights the difficulties associated with identifying highly degenerate repeat domains. More recently though, Ye et al. (2015) have demonstrated that WYL motifs have highly conserved α-helical secondary structures. Furthermore, the few amino acid residues that are conserved between such WYL or WY motifs have been shown to be hydrophobic, occupying buried positions within these α-helices (Boutemy et al., 2011; Chou et al., 2011; Win et al., 2012b; Ye et al., 2015). Thus, an integrated approach, combining HMM screens, together with secondary structure predictions and surface accessibility profiles, can be employed to identify the degenerate, and often repeated, WYL or WY motifs present in oomycete RXLR effectors.

## Repeat domains play diverse roles in RCP effector function

Collectively, repeat domains play diverse roles in the biological function of RCP effectors from plant-associated organisms (Tables 1–3). In brief, these roles can range from directing effector localization, to mediating interaction with one or more specific RNA, DNA, protein, or carbohydrate targets, to providing effector stability. It is becoming increasingly clear that these roles are intimately linked to the composition or architecture of the repeat domains that perform them. For example, as shown in Figures 1, 2, the repeat domain of an RCP effector, like that of many other RCPs (Grove et al., 2008), frequently exhibits an extended modular, non-globular architecture. This in turn provides the effector with a larger surface area-to-volume ratio than that of a typical globular protein of equivalent amino acid length, a feature that is particularly well-suited to certain functional roles. This is elegantly illustrated by the transcription activator-like (TAL) effectors of the bacterial plant pathogens, *Xanthomonas* spp., which interact with host DNA in the plant cell nucleus to hijack host genes (by transcriptional activation) whose expression promotes bacterial growth and/or disease symptom formation (Boch and Bonas, 2010). TAL effectors carry a central repeat domain that possesses up to 33.5 near-identical tandem repeats of 30–42 amino acids in length, followed by a carboxyl (C)-terminal region that contains both NLSs and a eukaryotic acidic activation domain (Boch and Bonas, 2010). As shown for PthXo1, a TAL effector from the rice blight pathogen, *Xanthomonas oryzae* pv. *oryzae*, the central repeat domain forms an extended surface area of interaction with host DNA, in which the repeat domain adopts an α-solenoid structure that physically wraps around the DNA molecule (Figure 1C) (Deng et al., 2012; Mak et al., 2012). More specifically, the individual repeat units mediate the direct binding of single consecutive nucleotide bases within the promoter sequence (i.e., the effector-binding element; EBE) of a host gene. This specificity is governed by amino acid residues 12 and 13 of each repeat unit, termed the repeat-variable di-residues (RVDs), which make specific contact with the host DNA and play a stabilizing role, respectively (Boch et al., 2009; Moscou and Bogdanove, 2009). The functional relevance of this repeat structure was reinforced by artificial TAL effectors carrying a variable number of repeat units. Boch et al. (2009) were able to show that a minimum of 6.5 repeat units are necessary for EBE recognition and subsequent transcriptional activation, while 10.5 or more repeat units are required for strong target gene expression.

An extended modular, non-globular architecture, as adopted by the repeat domains of many RCPs, is also particularly well-suited to mediating various protein–protein interactions (Grove et al., 2008). Indeed, many classes of repeat domains serve as scaffolds or adaptors. When performing this role, different repeat units, or regions of a repeat unit, may organize multiple proteins into functional complexes. Alternatively, interactions between different proteins, or between proteins and other functional domains present in the RCP, may be facilitated (Grove et al., 2008). Importantly, these roles are supported by the inherent conformational flexibility of the repeat domain, as mediated through for instance, a flexible hydrophobic core (Kappel et al., 2010), or flexible inter-repeat hinges, loops, or linkers, similar to those found in Cin1, a candidate effector of unknown function from the apple scab fungus, *Venturia inaequalis* (Figure 2C) (Mesarich et al., 2012). Domains that may perform such a role include, for example, those comprising ankyrin or HEAT/armadillo repeats, which, like the repeat domains present in TAL effectors, adopt an α-solenoid-type architecture, as well as leucine-rich repeats (LRRs), which adopt an α/β-solenoid-like or horseshoe-type fold (Kajava, 2012). Notably, several effectors from plant-associated organisms carry such repeat domains. For example, effectors of the bacterial wilt pathogen, *Ralstonia solanacearum*, including RipAP, RipBB, RipBC, and RipY, carry ankyrin repeats (Peeters et al., 2013), while other effectors of *R. solanacearum* and *Xanthomonas* spp., including RipS1–RipS8, XopAD, and XopN, carry HEAT/armadillo repeats (White et al., 2009; Peeters et al., 2013). In addition, several effectors from *R. solanacearum* (RipG1–RipG7), *Xanthomonas* spp. (XopAC, XopAE, and XopL), and the gall-forming pest of cereals, *Mayetiola destructor* (SSGP-71 family), carry LRRs (Figure 1D) (Xu et al., 2008; White et al., 2009; Peeters et al., 2013; Zhao et al., 2015).

Of the effectors mentioned above, one of the best characterized to date is XopN, a type III effector widely conserved across *Xanthomonas* spp. that suppresses host immune responses (Roden et al., 2004; Kim et al., 2009; Taylor et al., 2012). XopN from the leaf spot pathogen of pepper and tomato, *Xanthomonas euvesicatoria*, carries seven tandem HEAT/armadillo-like repeats (Roden et al., 2004). This effector interacts with the atypical LRR-receptor-like kinase (RLK), TARK1 (via the XopN non-repetitive N-terminal region), and the 14-3-3 isoform, TFT1 (via the XopN C-terminal HEAT/armadillo-like repeats), two positive regulators of host immunity in tomato, near and at the plant cytoplasmic–plasma membrane (PM) interface, respectively (Kim et al., 2009; Taylor et al., 2012). In addition to these binary interactions, XopN also engages in tertiary interactions with TARK1 and TFT1 at the plant cytoplasmic–PM interface (Kim et al., 2009; Taylor et al., 2012). Here XopN is expected to promote and/or stabilize TARK1/TFT1 complex formation by functioning as a protein bridge or molecular scaffold (Taylor et al., 2012). Currently however, it remains unclear how these interactions suppress host immune responses. One possibility is that XopN interferes with TARK1 protein–protein interactions, stability and/or signal transduction, and in the case of TFT1, client interactions (Kim et al., 2009; Taylor et al., 2012). Another possibility, given that TARK1 and TFT1 do not interact in the absence of XopN, is that the binding of this effector to these proteins in either binary or tertiary complexes leads to the sequestration of inactive immune complexes at or near the plant cytoplasmic–PM interface, thereby preventing downstream immune signaling (Taylor et al., 2012).

Other repeat domain architectures and compositions have been shown to play an important role in the function of RCP effectors from plant-associated organisms. One such example is provided by Ecp6, an effector of the tomato leaf mold fungus, *Cladosporium fulvum*, which carries three lysin motif (LysM) domains that each adopt a β*ααβ*-fold as part of an overall globular structure (Figure 2D) (Bolton et al., 2008; Sánchez-Vallet et al., 2013). Ecp6 molecules sequester chitin oligosaccharides released from the cell wall of *C. fulvum* during infection. In doing so, Ecp6 prevents the recognition of these oligosaccharides by host chitin immune receptors, thereby perturbing chitin-triggered immunity (de Jonge et al., 2010). More specifically, two of the three LysM domains, LysM1, and LysM3, undergo chitin-induced dimerization, in which the domains cooperate to produce a deeply buried chitin-binding groove (Figure 2D). This groove binds a single chitin oligosaccharide with ultra-high affinity, and is sufficient to out-compete host chitin immune receptors for chitin binding (Sánchez-Vallet et al., 2013). Another example is provided by GrCLE1, an effector of the potato cyst nematode, *Globodera rostochiensis* (Lu et al., 2009). GrCLE1 possesses a variable domain, followed by a C-terminal region with four 12-amino acid repeats that have similarity to plant CLAVATA3 (CLV3)/endosperm surrounding region (ESR)-related (CLE) peptides (Lu et al., 2009). In plants, endogenous CLE protein precursors are post-translationally modified and proteolytically processed to give bioactive CLE peptides. These peptides then function as hormones that interact with various extracellular plant receptors to regulate many aspects of plant growth and development (Kucukoglu and Nilsson, 2015). Like plant CLE protein precursors, GrCLE1 is post-translationally modified and proteolytically processed by plant machinery to produce bioactive CLE-like peptides (Guo et al., 2011; Chen et al., 2015). These peptides then function as endogenous plant CLE peptide mimics, directly binding plant RLKs, including CLV2, BAM1, and BAM2, to alter plant root growth and development for the promotion of plant parasitism (Lu et al., 2009; Guo et al., 2011; Chen et al., 2015).

## Several RCPs of plant-associated organisms are surface-associated

An important point to stress is that several RCPs of plant-associated organisms are surface-associated. That is, they are attached to, or are integrated into, the cell wall and/or PM through various covalent/non-covalent linkages or transmembrane domains, and are at least partially exposed to the extracellular environment. Although not classified as typical secreted effectors, a number of these surface-associated RCPs, and more specifically their repeat domains, have been shown or are hypothesized to play a role in interactions between plant-associated organisms and their hosts (e.g., Görnhardt et al., 2000; Robold and Hardham, 2005; Lanver et al., 2010; Pradhan et al., 2012). An example is provided by CBEL, a cell wall glycoprotein from *Phytophthora parasitica* var. *nicotianae* (*Ppn*), the oomycete root pathogen responsible for black shank disease of tobacco (*Nicotiana tabacum*) (Séjalon-Delmas et al., 1997; Villalba Mateos et al., 1997). CBEL possesses two repeats, each comprising a carbohydrate-binding module family 1 (CBM1)/fungal-type cellulose-binding domain (CBD) attached to a PAN/APPLE domain (Séjalon-Delmas et al., 1997; Villalba Mateos et al., 1997). Functional analyses have determined that these CBDs play a role in the adhesion of *Ppn* mycelia to cellulosic substrates, including plant cell walls, and in the organized deposition of the *Ppn* cell wall polysaccharide, β-glucan (Villalba Mateos et al., 1997; Gaulin et al., 2002, 2006). Interestingly, CBEL also elicits strong host immune responses when infiltrated into tobacco (Villalba Mateos et al., 1997), as well as various non-host plants, including *A. thaliana* (Khatib et al., 2004; Gaulin et al., 2006). These responses are dependent upon the binding of CBEL to the plant cell wall, as mediated through the CBDs (Gaulin et al., 2006). A second example is provided by Rep1 of the corn smut fungus, *Ustilago maydis*, which carries 12 mostly tandem repeats of 34–55 amino acids in length (Wösten et al., 1996). These repeats, which carry Kex2 recognition sites, are processed in the secretory pathway to 11 repellent peptides that form rigid surface-active amyloid-like fibrils at the hyphal surface, and play a role in cellular attachment to hydrophobic surfaces (e.g., the plant surface) and in the formation of aerial hyphae (Wösten et al., 1996; Teertstra et al., 2006, 2009; Müller et al., 2008; Lanver et al., 2014).

## Repeat domains may contribute to the adaptive evolution of RCP effectors

Repeat domains can evolve in several different ways, including through changes in repeat unit number or order, as well as through amino acid substitutions or insertions/deletions (indels) in repeat units and/or associated interconnecting loop/linker regions. Changes in number or order, particularly for those repeat units encoded by long nucleotide sequences (≥10 nucleotides in length), likely evolve through intra- and inter-genic recombination events (Richard and Pâques, 2000). As shown in other systems, the mutation rates associated with these changes can be orders of magnitude greater than those associated with point mutations, accelerating the evolution of the coding sequence to which they belong (reviewed in Gemayel et al., 2010). Indeed, repeat unit number and/or order has commonly been shown to vary between RCP effectors and RCP effector candidates of individuals, strains, or isolates of the same species or pathovar of plant-associated organism (e.g., Allen et al., 2004; Heuer et al., 2007; Jelenska et al., 2007; Kucheryava et al., 2008; Aggarwal et al., 2014). Changes in repeat unit number have also been shown to accompany the evolutionary paths of certain effector families from plant-associated organisms (e.g., Goss et al., 2013). Furthermore, chimeric RCP effectors, resulting from a recombination event between homologous repeat domains, have been reported (e.g., Yang et al., 2005), a finding that is not surprising, given the high number of RCP effectors that belong to multi-protein families (Tables 1–3). Although generally not as quick to accumulate, amino acid substitutions, and indels also play an important role in generating sequence diversity within a repeat domain. However, these types of modification only occur following a duplication event. Again, such sequence variation has commonly been found to occur between the repeat units of RCP effectors or RCP effector candidates (see imperfect or degenerate repeat units listed in Tables 1–3), as well as between the repeat domains of RCP effectors and RCP effector candidates from individuals, strains, or isolates of the same species or pathovar of plant-associated organism (e.g., Kucheryava et al., 2008; Chou et al., 2011; Ve et al., 2013).

Of what relevance could this repeat domain variability be to plant-associated organisms? In industrial and animal-pathogenic yeasts, alterations to the repeat unit number, and/or order of surface-associated RCPs, termed adhesins, have been shown to impart changes in adhesion phenotype, which may permit the rapid adaptation of these organisms to different substrates and host tissues, respectively (reviewed in Verstrepen and Fink, 2009). Furthermore, variability in the repeat domains of RCPs has been linked to the evasion of host immune responses in animal systems (e.g., Madoff et al., 1996; Mendes et al., 2013). In plant-associated organisms, the first indication that repeat domain variability could confer RCP effectors with an adaptive advantage, by providing a source of functional diversity, flexibility, and/or a means of evading host recognition, was provided by the experimental manipulation of AvrBs3, a TAL effector from *X. euvesicatoria* (Herbers et al., 1992). Typically, in a compatible interaction with pepper plants, AvrBs3 transcriptionally activates *UPA20*, a host gene that encodes a basic helix-loop-helix transcription factor, to trigger plant cell hypertrophy (Marois et al., 2002; Kay et al., 2007). However, in an incompatible interaction, AvrBs3 transcriptionally activates *Bs3*, a pepper gene that encodes an executor resistance protein with homology to flavin monooxygenases, to trigger host immunity (Römer et al., 2007, 2009). To dissect the molecular basis of *Bs3*-dependent immunity, Herbers et al. (1992) generated random deletion derivatives of AvrBs3 that differed in their repeat unit number. While most AvrBs3 deletion derivatives lost their ability to trigger *Bs3*-dependent immunity, others gained a new host specificity, triggering immunity in pepper plants carrying *Bs3-E*, an allele of *Bs3* (Herbers et al., 1992). This research, which was subsequently confirmed by repeat domain swaps between other TAL effectors (e.g., Yang et al., 2005), demonstrated that it is the order, and thus the sequence, of TAL repeat units that determines host specificity. In addition, this research raised the possibility that recombination within or between the repeat domains of TAL effectors could produce novel effectors capable of activating different host genes (and thus promoting different host interaction phenotypes) as a consequence of their altered DNA recognition specificities. Indeed, evidence for inter- and intra-genic recombination events between TAL effectors has since been provided (Yang and Gabriel, 1995; Yang et al., 2005).

Aside from those present in TAL effectors, other repeat domains have been implicated in the adaptive evolution of RCP effectors from plant-associated organisms. An example is provided by the hypervariable (Gp-HYP) effectors of the potato cyst nematode, *Globodera pallida*, which are targeted to the host apoplast throughout biotrophy, and are required for successful root colonization (Eves-van den Akker et al., 2014). Gp-HYP effectors, which possess several conserved regions and a central repeat domain, are encoded by a large and incredibly complex gene family. Based on repeat domain amino acid sequence, these effectors can be assigned to one of three subfamilies (Gp-HYP-1, -2, and -3), with members of Gp-HYP-1 and -3 demonstrating high variability in the number, sequence, and order of their tandem repeats (Eves-van den Akker et al., 2014). Notably, *Gp-HYP* genes exhibit unparalleled diversity between individuals of the same population, with no two nematodes possessing the same genetic complement of *Gp-HYP-1* and *-3* genes. While it remains unclear what functional role the Gp-HYP repeat domains play in the context of plant parasitism by *G. pallida*, it has been suggested that their variability may reflect functional diversity, possibly in specificity of ligand binding. It has also been suggested that this variability may reflect the need to evade host recognition, possibly providing an explanation as to why breeding broad-spectrum resistance against this nematode has been so difficult (Eves-van den Akker et al., 2014). In another example, it has been suggested that the duplication and subsequent sequence diversification of CLE-like repeats present in the GrCLE effectors of *G. rostochiensis* may represent an important mechanism for generating functional diversity required for host parasitism. This is based on the finding that the ectopic over-expression of different GrCLE RCP effectors in *A. thaliana* leads to a wide range of plant phenotypes (Lu et al., 2009).

For several RCP effectors, including ATR1 of *H. arabidopsidis* (and other RXLR effectors from plant-pathogenic oomycetes), as well as AvrM-A of *M. lini*, and AvrPtoB of *Pst*, sequence diversification has been shown to play a particularly important role in driving repeat domain evolution, with the repeat units present in these effectors lacking significant amino acid sequence homology (Jiang et al., 2008; Dong et al., 2009; Chou et al., 2011; Ve et al., 2013). Instead, typically only those amino acid residues required for maintenance or stabilization of the overall tertiary fold or structural core have remained conserved or physicochemically similar between repeat units (Cheng et al., 2011; Chou et al., 2011; Ve et al., 2013). This in turn has provided these effectors with a conserved structural framework for rapid diversification, a feature that may promote functional diversity, flexibility, and/or a means of evading host recognition. Certainly, the repeat units of AvrPtoB provide an excellent example of functional flexibility. As mentioned previously, the N terminus and central region of this effector each carry a single repeat unit that adopts a four-helix bundle fold (repeat units one and two, respectively; Figures 1A,B), while the C terminus carries a U-box-type E3 ubiquitin ligase domain (Abramovitch et al., 2006; Janjusevic et al., 2006; Dong et al., 2009; Cheng et al., 2011). Remarkably, both repeat units play distinct and multiple roles in modulating host immune responses. For example, repeat units one and two bind and inhibit the kinase domain of the PM-localized host LysM-RLK and LRR-RLK immune receptors, Bti9 and BAK1, respectively, to suppress immunity-related signaling (Göhre et al., 2008; Shan et al., 2008; Cheng et al., 2011; Zeng et al., 2012). Repeat units one and two also bind the kinase domain of the LysM-RLK CERK1 and LRR-RLK FLS2 immune receptors, respectively, which may promote their ubiquitination and subsequent proteasome-dependent degradation via the AvrPtoB E3 ligase domain (Göhre et al., 2008; Gimenez-Ibanez et al., 2009). In addition, repeat unit one interacts with the host receptor-like cytoplasmic kinase (RLCK) Pto, while repeat unit two interacts with Pto and a related host RLCK, Fen (Rosebrock et al., 2007; Dong et al., 2009; Mathieu et al., 2014). Of note, in line with the observed sequence diversity, structural analyses have determined that repeat unit one interacts with the Pto kinase in a different orientation to that of repeat unit two with the BAK1 kinase domain (Figures 1A,B) (Dong et al., 2009; Cheng et al., 2011). Interestingly, in conjunction with Prf, an immune receptor of tomato, Pto is able to activate host immunity following its interaction with AvrPtoB (Kim et al., 2002; Mucyn et al., 2006; Dong et al., 2009). Fen however, can only activate host immunity in the absence of the E3 ubiquitin ligase domain (Rosebrock et al., 2007). It has now been shown that interaction of either Pto or Fen with repeat unit two results in the proteasome-dependent degradation of these RLCKs as above (Rosebrock et al., 2007; Mathieu et al., 2014). Pto however, is able to resist AvrPtoB-mediated degradation and activate Prf-dependent immunity following its interaction with repeat unit one, as this repeat unit is further away from the E3 ubiquitin ligase domain (Mathieu et al., 2014). It has been suggested that Pto and Fen evolved as decoys of the aforementioned non-cytoplasmic kinases to provide immunity against *Pst* (Block and Alfano, 2011).

## Conclusion and perspective

Analyses of protein sequence and tertiary structure have revealed that several effectors of plant-associated organisms are RCPs. As reviewed here, repeat domains play diverse roles in RCP effector function. Furthermore, repeat domains may contribute to the rapid adaptive evolution of RCP effectors, providing a source of functional diversity, flexibility, and/or a means of evading host recognition. With these points in mind, it is perhaps not surprising that increased attention has been given to the identification of RCP effectors from plant-associated organisms (e.g., Mueller et al., 2008; Raffaele et al., 2010; Rudd et al., 2010; Saunders et al., 2012; Rafiqi et al., 2013). Undoubtedly, as (1) more genomes of plant-associated organisms are sequenced; (2) the tools of repeat identification become more powerful; and (3) additional effectors are structurally characterized, many more RCP effectors will be identified. The ongoing challenge will be to understand the precise roles that repeat domains play in the function and adaptive evolution of these effectors. Curiously, many of the repeat domain classes discussed in this review are also co-opted by plants to mediate ligand recognition and/or signaling associated with symbiosis, immunity, as well as physiology and development (Palma et al., 2005; Wang et al., 2006; Laluk et al., 2011; Gust et al., 2012; Böhm et al., 2014; Cui et al., 2015; Kucukoglu and Nilsson, 2015). Thus, as shown for the CLE-like repeats of GrCLE1 from *G. rostochiensis* (Lu et al., 2009; Guo et al., 2011), it is likely that many RCP effector repeat domains mimic host components associated with these processes to facilitate colonization.

Although not discussed in this review, we acknowledge that repeat domains can be intrinsically disordered (ID); a feature characterized by conformational flexibility and a lack of secondary or tertiary structure under physiological conditions (Dyson and Wright, 2005). In fact, repetitive sequence, along with a preponderance of charged and hydrophilic amino acid residues, is often a hallmark of ID (Dyson and Wright, 2005). Like the ordered (structured) repeat domains described above, ID regions carry out diverse roles in protein function, ranging from providing a flexible linker between structured domains, to mediating protein–protein interactions (Dyson and Wright, 2005). To date, examples of RCP effectors with such a repeat domain architecture remain limited, although ID has been predicted for the P/Q-rich repeats of HopI1, a type III effector from the Brassicaceae leaf spot pathogen, *P. syringae* pv. *maculicola* (Table 1; Jelenska et al., 2010; Marín and Ott, 2014). Of relevance, many ID regions are known to undergo induced folding upon interaction with their physiological targets, a process that gives rise to the unusual combination of low affinity and high specificity, which may allow these interactions to be readily reversible or may confer flexibility and promiscuity to target binding (Dyson and Wright, 2005). Furthermore, likely owing to a lack of structural constraints, ID protein sequences often evolve at a faster rate than ordered protein sequences, acquiring a greater number of single amino acid substitutions, insertions, deletions, and repeat unit expansions (Brown et al., 2011; Nilsson et al., 2011). Consequently, ID repeat domains are also of great interest to understanding how RCP effectors circumvent host recognition, or acquire novel, altered, and extended effector functionalities that further enhance the colonization of susceptible hosts (Marín et al., 2013; Marín and Ott, 2014).

## Author contributions

CM, JB, and MT conceived the review. CM wrote the manuscript. CM and CH prepared Figures 1, 2. CM and JB constructed Tables 1–3. CM, JB, CH, and MT critically revised the manuscript. All authors approved the final version of the manuscript.

### Conflict of interest statement

The authors declare that the research was conducted in the absence of any commercial or financial relationships that could be construed as a potential conflict of interest.
